# Chemical Aristocracy: He_3_ Dication and Analogous Noble-Gas-Exclusive Covalent Compounds

**DOI:** 10.1021/acs.jpclett.4c00826

**Published:** 2024-03-29

**Authors:** Lucas Araujo, Felipe Fantuzzi, Thiago M. Cardozo

**Affiliations:** † Instituto de Química, Universidade Federal do Rio de Janeiro, Av. Athos da Silveira Ramos 149, Rio de Janeiro 21941-909, Brazil; ‡ School of Chemistry and Forensic Science, University of Kent, Park Wood Road, Canterbury CT2 7NH, U.K.

## Abstract

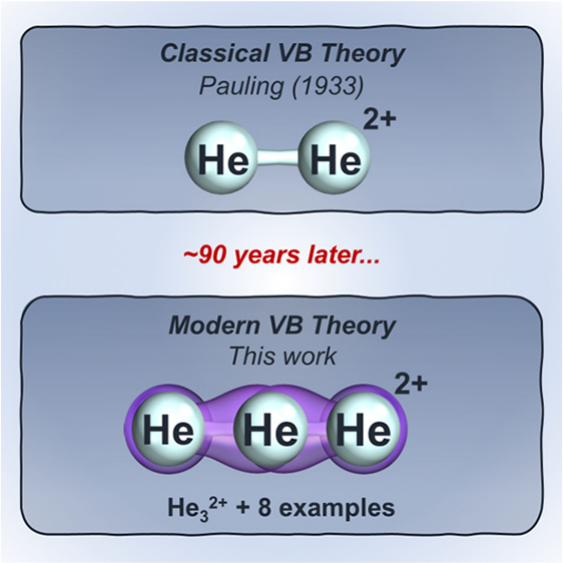

Herein, we predict the first set of covalently bonded triatomic molecular compounds composed exclusively of noble gases. Using a combination of double-hybrid DFT, CCSD(T), and MRCI+Q calculations and a range of bonding analyses, we explored a set of 270 doubly charged triatomics, which included various combinations of noble gases and main group elements. This extensive exploration uncovered nine noble-gas-exclusive covalent compounds incorporating helium, neon, argon, or combinations thereof, exemplified by cases such as He_3_^2+^ and related systems. This work brings to light a previously uncharted domain of noble gas chemistry, demonstrating the potential of noble gases in forming covalent molecular clusters.

Noble gases are well-known as the most inert group of elements in the periodic table. Nevertheless, their inertness can be overcome, depending on their chemical environment, resulting in the formation of both stable and metastable compounds. Helium, for example, has been observed to form a stable compound with sodium under high-pressure conditions,^1^ while argon can form species such as HArF in cold matrices^2^ and organometallic compounds under supercritical noble gas solutions.^3^ Moreover, HeH^+^^4−6^ is posited as the first molecule to be formed in the universe, and its presence in interstellar environments was confirmed after its unambiguous detection in the planetary nebula NGC 7027. Other noble gas hydrides, such as HeH_2_^+^^7^ and ArH^+^,^8^ are also pointed out as key players in galactic and extragalactic regions. These studies underscore the richness of gas-phase ion chemistry within the realm of noble gases, which has been the subject of numerous investigations and research efforts.^7,9−12^

Molecular dications are intriguing species that can manifest in a variety of environments, particularly in ionizing and low-pressure settings, such as those found in upper-planetary atmospheres and the interstellar medium. These entities have also garnered attention for their potential role in propulsion systems.^13^ While diatomic molecular dications containing noble gases, especially those containing helium, have been extensively investigated,^14,15^ the exploration of dications with more than two atoms has been relatively limited. Among these systems (see Figure 1A), structures analogous to acetylene containing helium (HeCCHe^2+^) and neon (NeCCNe^2+^) are noteworthy.^16,17^ More recently, novel CH_4_Ng^2+^ (Ng = He–Rn) dications resembling methanium (CH_5_^+^) and showcasing strong C–Ng bonds have been proposed.^18^ The design of these systems usually involves replacing a hydrogen atom with Ng^+^ or, alternatively, substituting a hydrogen ion with Ng^2+^ within the molecular structure. The inherent challenges of conducting experimental studies in helium molecular ion chemistry, coupled with the computational tractability of these small-sized compounds, highlight the paramount significance of theoretical investigations in furthering our comprehension and predictive potential within this domain.^19^

**Figure 1:**
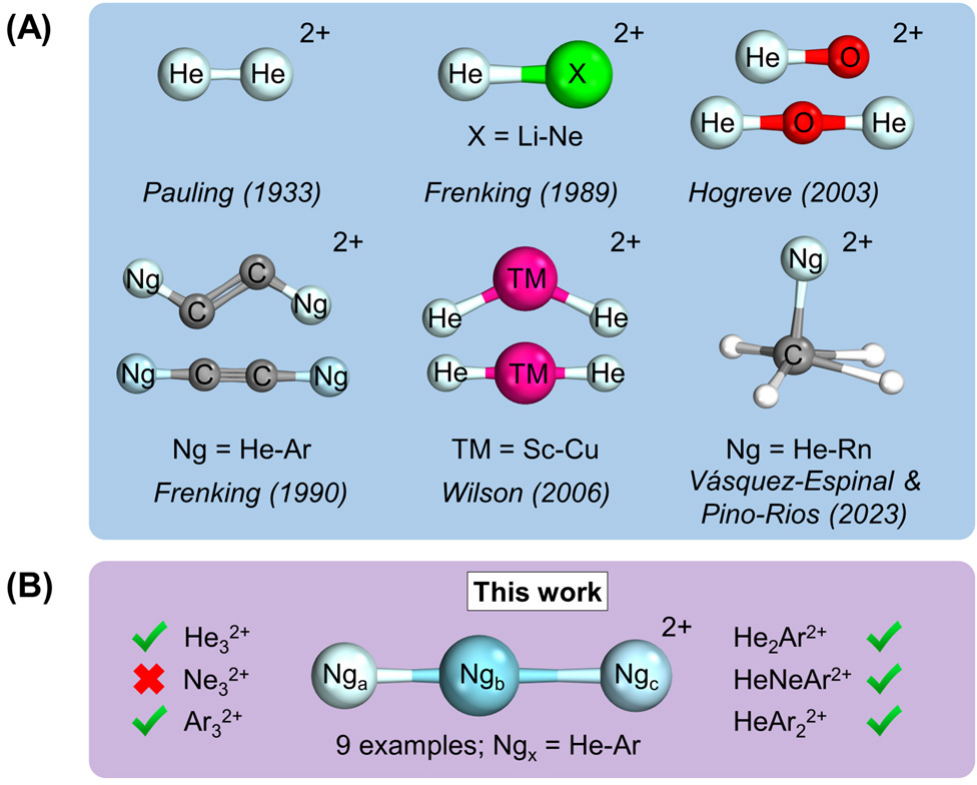
Predicted molecular dications featuring light noble gases. (A) Selected literature examples of (meta)stable dicationic molecular systems featuring noble gases, especially helium. (B) Noble-gas-exclusive compounds were predicted in this work.

The resistance of noble gases to form chemical bonds, especially when electrically neutral, is not surprising. These elements possess exceptionally high excitation energies and exhibit the highest ionization potentials (IPs) within the periodic table. The main challenge in dealing with noble gases lies in generating atomic electronic configurations that are conducive to the formation of chemical bonds. Fluorine, characterized by its remarkably high IP, can offset the excitation energy of noble gas electronic configurations, allowing for the formation of noble-gas fluorides. Indeed, species such as KrF_2_, XeF_2_, and RnF_2_ are among the most renowned compounds involving noble gases.^20^ Notably, they represent a rare category of symmetric insertion compounds with noble gases, with RnF_2_ being the only radon compound experimentally reported. Ionized noble gases can form surprisingly stable compounds given that they lack the stable closed-shell configuration of their neutral counterparts.^21,22^

Noble-gas insertion compounds, typically represented as X-Ng-Y, constitute an exceedingly rare category of molecules, especially in the case of the lighter Ng atoms. Experimental reports of species with He or Ne insertions remain conspicuously absent.^23^ The quest for lighter noble gas insertion compounds therefore presents a formidable challenge. In the case of helium, obtaining these compounds signifies reaching its maximum valency, which is a fascinating chemical pursuit. To address this, charged insertion complexes seem to offer a promising starting point. Most of the reports so far present structures of the form H-Ng-L^+^, where L represents ligands like CO,^24^ CCO,^25^ CS,^26^ BF,^27^ OSi,^28^ and N_2_.^29^ Helium, for example, can form metastable anions with a FHeX^–^ structure (X = O, S, Se, CC).^30−33^ Additionally, noble gas complexes commonly exhibit fluorine, prompting an ongoing quest for fluorine-free compounds.^34^ Investigations into dicationic complexes have largely focused on diatomic^15,35^ and triatomic molecules^16,36,37^ with at most two noble gas atoms.

During an extensive exploratory investigation focusing on multiply charged species, we meticulously analyzed a set of 270 doubly charged triatomic molecules characterized by a closed-shell configuration and the general form [X–Ng–Y]^2+^ where X and Y = H–Ar and Ng = He, Ne, or Ar. To accomplish this, we combined double-hybrid density functional theory (DFT) at the DSD-BLYP/aug-cc-pVTZ level, coupled cluster calculations based on the CCSD(T)/aug-cc-pVTZ level, additional wave function methods, and a variety of bonding analyses. Our discussion centers on our intriguing findings arising from sets of molecules composed exclusively of noble gases. Surprisingly, many of the noble-gas-exclusive systems revealed remarkably short Ng–Ng chemical bonds, with atoms arranged in a perfectly linear configuration. These systems can be formally described as Ng insertion compounds with Ng terminal atoms. Table S1 in the Supporting Information provides a comprehensive listing of all predicted structures and their corresponding bond lengths across the various levels of theory considered.

We begin our discussion with a concise overview of the chemically bonded structures uncovered in our investigation. From a pool of 18 prospective candidates, we have identified nine hitherto unknown compounds exclusively composed of noble gases, incorporating atoms spanning from He to Ar (Figure 1B). These compounds are identified as He_3_^2+^, He_2_Ar^2+^, HeNeAr^2+^, HeArHe^2+^, HeArNe^2+^, HeAr_2_^2+^, NeArNe^2+^, NeAr_2_^2+^, and Ar_3_^2+^. A noteworthy observation is that He_2_Ne^2+^ and NeHeAr^2+^ were established as minimum-energy structures when scrutinized through the DFT and MP2 approaches. However, this conclusion was not upheld at the CCSD(T) level, and the systems were therefore removed from our set. Among the nine compounds found, of particular significance are He_3_^2+^, HeArHe^2+^, NeArNe^2+^, and Ar_3_^2+^. These systems represent symmetric insertion compounds, an exceedingly rare category within the realm of noble gas compounds. It is also worth highlighting that Ne_3_^2+^ did not exhibit a minimum-energy configuration at any computational level, while well-defined potential energy wells for He_3_^2+^ and Ar_3_^2+^ were successfully characterized.

We first turned our attention to He_3_^2+^, the lightest among our investigated compounds. This linear molecule is characterized by having high vibrational frequencies, ranging from 942.94 cm^–1^ for the degenerate angular distortion, 1169.79 cm^–1^ for the symmetric stretching, and 2207.86 cm^–1^ for the asymmetric stretching at the CCSD(T)/aug-cc-pVTZ level of theory. The doubly charged helium dimer, He_2_^2+^, was originally predicted by Pauling in 1933^38^ and observed around 50 years later.^39^ The bond lengths within He_3_^2+^ are shorter than in He_2_^+^ but longer than in He_2_^2+^, and this correlates with the sum of the covalent radii.^40^ To the best of our knowledge, the existence of a triatomic helium dication has never been reported before. Figure 2A illustrates the potential energy curve for He_3_^2+^. Due to its multireferential nature, especially in regions distant from the energy minimum, we conducted an MRCI+Q (multireference configuration interaction with Davidson correction)^41,42^ scan using full CASSCF (complete active space self-consistent field)^43,44^ wave functions. The scan clearly reveals a metastable profile with an energy barrier of 16.1 kcal mol^–1^, a characteristic associated with chemical species that exhibit lifetimes on the order of hundreds of seconds.^45,46^ We also performed calculations for this particular molecule using the AQCC (averaged quadratic coupled cluster)^47^ method and the aug-cc-pVQZ basis set for benchmarking. Table S2 presents all vibrational frequencies of all of the predicted molecules.

**Figure 2:**
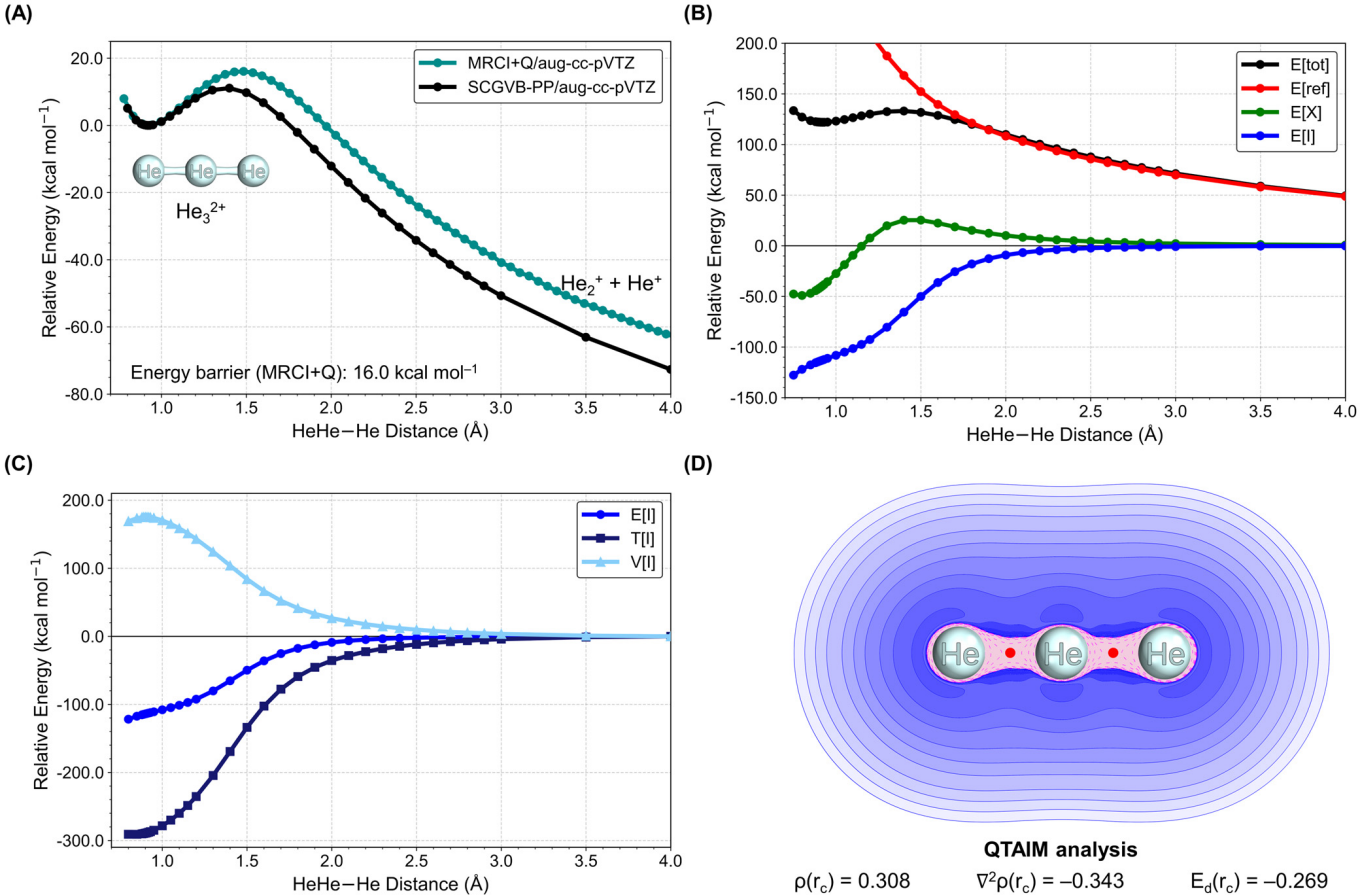
Quantum chemical analyses of He_3_^2+^. (A) Potential energy curve for the dissociation of one He–He bond in He_3_^2+^ at the MRCI+Q/aug-cc-pVTZ and SCGVB/aug-cc-pVTZ levels of theory. The energy values are relative to those of the optimized He_3_^2+^ structure at the CCSD(T)/aug-cc-PVTZ level. (B) Interference energy analysis (IEA) for He_3_^2+^, with E[ref] being the quasi-classical energy, E[I] the covalent term due to interference, and E[X] the exchange energy between electrons in different bonds. All curves are constructed relative to a 4.0 Å separation between He^2+^ and He^+^. (C) Interference energy partitioning into kinetic (T[I]) and potential (K[I]) contributions, according to IEA. (D) Plot of the Laplacian of the electron density (∇^2^ρ) in He_3_^2+^ at the CCSD(T)/aug-cc-pVTZ level. Blue corresponds to ∇^2^ρ > 0 and pink to ∇^2^ρ < 0. Red dots correspond to the bond critical points (BCPs). Selected QTAIM topological properties (au) at the BCPs, namely, ρ(r_c_), ∇^2^ρ(r_c_), and E_d_(r_c_), are also shown.

In order to shed light on the bonding structure of He_3_^2+^ and gain deeper insights into the nature of the chemical bond within this molecule, we performed calculations based on the spin-coupled generalized valence bond (SCGVB) theory.^48−50^ The similarity of the MRCI+Q and SCGVB approaches for He_3_^2+^ is clearly evident upon comparison of the HeHe–He potential energy curves of the ground state at both the MRCI+Q/aug-cc-pVTZ and SCGVB-PP/aug-cc-pVTZ levels. Here, PP denotes the perfect-pairing approximation, and the comparison between the curves is graphically represented in Figure 2A.

Leveraging the SCGVB description of the potential energy curve of He_3_^2+^, we assessed the covalent nature of the He–He chemical bond in this compound using the interference energy analysis (IEA),^51,52^ whose main results are shown in Figure 2A–C. This analysis is grounded in the generalized product function energy partitioning (GPF-EP) method,^51−53^ as outlined in the Supporting Information. Briefly, the GPF-EP approach adapts, for an SCGVB-type wave function, Ruedenberg’s partitioning scheme of the electron density—and, consequently, the electronic energy—into quasi-classical and quantum interference components, with the latter primarily linked to covalent effects.^54,55^ The quasi-classical components are denoted as reference (E[ref]), the exchange between electrons on different electron groups is termed (E[X]), while the first- and second-order interference energy terms are labeled as E[I] and E[II], respectively. It is worth mentioning that the E[II] term is negligible in the potential energy curves investigated herein, and therefore, only the E[I] term is shown.

Figure 2B provides clear insights into the origin of the metastable minimum in He_3_^2+^, which is primarily attributable to the E[I] term, contributing approximately 100 kcal mol^–1^ at the system’s equilibrium distance. This contribution closely resembles what was observed in our prior study of He_2_^2+^^56^ and unequivocally points to the covalent nature of the chemical bonds in He_3_^2+^. In an independent particle model (IPM) framework, covalent bonding arises from interference between orbitals. Energetically, quantum interference effectively reduces the electron kinetic energy (T) during bond formation. The reduction in kinetic energy caused by interference, denoted as T[I], is accompanied by an increase in the potential energy (V) caused by this effect with the corresponding quantity labeled as V[I]. This process is a hallmark of bond formation across various chemically bonded systems, as evidenced by a substantial body of research.^56−68,50^ In essence, quantum interference acts to redistribute electronic density, concentrating it more in the bonding region and thereby facilitating the bond formation. This pattern is clearly illustrated in the case of He_3_^2+^, as shown in Figure 2C. The behavior of T[I] and V[I] in He_3_^2+^ is consistent with the general trend observed in covalent bonds, reinforcing the critical role of quantum interference in bond formation.

Concurrently, E[ref] exhibits behavior akin to Coulomb repulsion, while E[X] between electrons in the different bonds, which stabilizes energies near the equilibrium distance, subsequently transforms into a destabilizing term, reaching its peak at the position of the energy barrier. All in all, the IEA results strongly affirm the presence of relatively robust He–He bonds in He_3_^2+^, sufficiently potent to overcome the substantial Coulombic repulsion imposed by the double charge. This results in the formation of a distinct metastable minimum, thereby expanding the possibilities for its successful detection, whether in controlled matrix isolation experiments or within the complex landscapes of interstellar environments.

In addition to the bonding analyses provided by inspection of the IEA curves, we have employed the topological analysis of quantum theory of atoms in molecules (QTAIM)^69,70^ to investigate the electron density on the calculated dications. Figure 2D showcases the Laplacian of the electron density for He_3_^2+^, along with the selected QTAIM properties. Similar data for the other noble-gas-exclusive systems can be found in the Supporting Information. Negative regions of the Laplacian of the electron density calculated at the bond critical point (BCP), ∇^2^ρ(r_c_), serve as indicators of the electron density concentration.

It is evident that for He_3_^2+^, the electron density is predominantly concentrated between the nuclei, leading to the emergence of distinct BCPs. A closer examination of the QTAIM descriptors at these BCPs reveals significant characteristics: a positive electron density value, ρ(r_c_), measuring +0.308, a negative ∇^2^ρ(r_c_) value at −0.343, and a negative local energy density, E_d_(r_c_), totaling −0.269. These values collectively reinforce the strong covalent nature of the bonds in He_3_^2+^,^71^ corroborating the findings obtained through the IEA approach.

We now turn our attention to the results of heavier Ng-exclusive dicationic triatomics investigated herein and compare them to the results obtained for He_3_^2+^. Charge distribution plays an important role in understanding the bonding in the predicted dications. The atomic charges of the molecular structures computed herein are depicted in Figure 3 and are also detailed in Table S3. Typically, charges tend to concentrate in elements with larger atomic volumes and therefore larger polarizabilities or at the extremes, where Coulomb repulsion is minimized.

**Figure 3:**
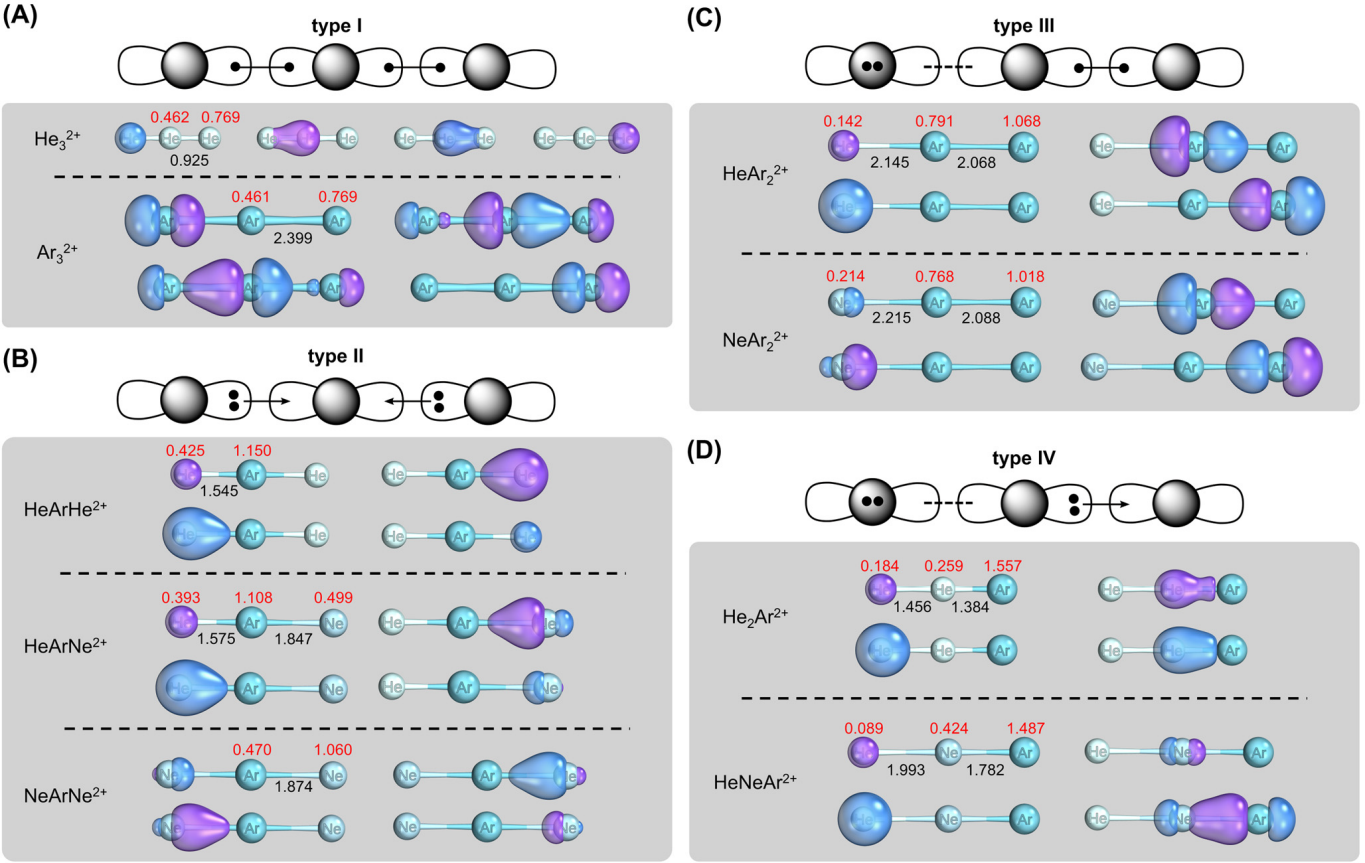
Four bonding patterns observed for the noble-gas-exclusive molecular dications and their corresponding SCGVB orbitals. (A) Type I bonding scheme, characterized by covalent bonds connecting all three atoms, followed by the SCGVB orbitals of the corresponding type I species. (B) Type II bonding scheme, featuring an electron-deficient central atom that forms donor–acceptor bonds with neighboring atoms and accompanied by SCGVB orbitals of the corresponding type II species. (C) Type III bonding scheme, where the central atom simultaneously forms a covalent bond with one atom and a noncovalent complex with another. SCGVB orbitals of the corresponding type III species are also shown. (D) Type IV bonding scheme, where the central atom engages in a donor–acceptor bond with one atom and a noncovalent complex with another, along with the corresponding SCGVB orbitals. In each case, the bond lengths (black) and ChelpG charges (red), computed at the CCSD(T)/aug-cc-pVTZ level, are shown. The bonding schemes are based on the computed SCGVB orbitals and Laplacians of the electron density.

The four one-electron full valence SCGVB orbitals for He_3_^2+^ in the CCSD(T) optimized geometry are shown in Figure 3A. At the ends, the helium atoms exhibit spherical s-like one-electron orbitals, while the central helium atom possesses highly polarized lobe orbitals oriented toward the ends, facilitating the formation of the chemical bonds and reducing the charges at the extremes. This leads to a separation of charges within the molecule, and the central noble gas orbitals become polarized to mitigate electrostatic repulsion, ultimately explaining the concentration of charges at the extremes. These orbitals display strong polarization toward the centers of charge, a characteristic somewhat reminiscent of the He_3_^2+^ case, albeit with distinct polarization patterns.

Figure 3 delineates a general model depicting the orbital polarization patterns across all molecules that exhibit true minima at the CCSD(T) level. Bonding is induced through strong polarization of valence orbitals by the charge centers in all cases. Figure 3A,B illustrates type I and type II patterns where atoms are held together solely by covalent interactions. Notably, in type I molecules, which are homonuclear in this context, a significant portion of the positive charge is concentrated over the outermost atoms, minimizing repulsion effects. The central atom is actively engaged in covalent bonds with the outer atoms. On the other hand, in type II, argon serves as the central atom, and almost all positive charge is distributed across its atomic volume. The electron density of the outer atoms is displaced toward the center to form coordinate bonds. In Figure 3C, we observe minima where argon atoms are engaged in covalent bonding, retaining most of the positive charge. Helium or neon atoms, in contrast, are bonded with the argon moiety through noncovalent interactions. The final scenario, illustrated in Figure 3D, demonstrates that an argon atom at one of the extremes retains most of the positive charge, inducing helium and neon to form a donor–acceptor bond directly. However, a third atom at the other extreme is only bonded through charge-induced noncovalent interaction, akin to the preceding case.

We have previously employed SCGVB orbitals in the description of highly polarized bonds.^72^ In Figure 3, we also present, in a simplified manner, orbital schemes representing the main interatomic interactions observed in the obtained minima. Lobe-type orbitals are used to represent orbitals that become polarized in the structures. Additionally, less polarized orbitals such as 1s orbitals are depicted as circles. Doubly occupied orbitals in the representation correspond to orbitals that are very similar after variational optimization. Arrows are employed to represent covalent dative bonds, and lines denote covalent two-center two-electron bonds; strong dashed lines indicate intermolecular interactions, such as ion-dipole interactions. Notably, the orbitals of atoms with lower charges are polarized toward the charge centers. This tendency is also evident upon analyzing the Laplacian of the electron densities and through intrinsic bond orbitals (IBOs).^73,74^ While IBOs have a limitation due to the double occupation of orbitals compared with VB calculations, they still provide valuable qualitative insights into the overarching bonding patterns. These bonding patterns are reflective of the charge distribution within the complexes, and Table S2 in the Supporting Information assigns each structure to one of the four types illustrated in Figure 3. Comprehensive data on the VB orbitals, Laplacians, and IBOs of all CCSD(T) minimum-energy structures are also available in the Supporting Information. Based on the calculated bond lengths, the empirical bond lengths, and the QTAIM properties, we proposed an assignment from the nature of the interactions. This assignment also corroborates with the VB orbitals associated with the molecules.

In summary, our study contributes to a growing body of literature that challenges the traditional view of noble gases as inert,^75^ revealing their potential in forming unique covalent molecular clusters. Using advanced computational methods including double-hybrid density functional theory and CCSD(T)/aug-cc-pVTZ calculations, along with a suite of additional wave function methods and bonding analyses, we have systematically explored a diverse set of 270 doubly charged triatomics. This exploration has led to the discovery of nine noble-gas-exclusive covalent compounds with metastable minima, involving helium, neon, argon, and their combinations. Our findings demonstrate further evidence that the presumed inertness of noble gases is not an unalterable truth but rather a conditional state dependent on structural and environmental factors. The remarkable ionization potentials of ionized noble gases allow them to induce nucleophilic behavior even in elements as inert as neutral helium and neon. This ability to polarize their own electronic densities has resulted in the formation of unexpected structures with distinct chemical bonds. This study opens a new horizon in noble gas chemistry, providing fresh perspectives on the bonding capabilities of noble gases. It also introduces potential candidates for detection in matrix isolation experiments and within ionizing and helium-rich environments commonly found in the interstellar medium.

## Data Availability

The data supporting the findings of this study are available in the paper and its Supporting Information, which includes additional calculations. Output files can be obtained from the corresponding authors upon reasonable request.

## Supporting Information Available

Details about the GPF-EP method; optimized geometries; calculated charges at the DFT, MP2, and CCSD(T) levels of theory; interference energy analysis; Laplacian of the electron density; and IBOs (PDF)
