# Supplementary material for: Chemical Aristocracy: He3 Dication and Analogous Noble-Gas-Exclusive Covalent Compounds
Source: J Phys Chem Lett. 2024 Mar 29;15(14):3757–63. doi: 10.1021/acs.jpclett.4c00826 (PMC11017316; doi:10.1021/acs.jpclett.4c00826)
Supplement: Supplementary file 1 — jz4c00826_si_001.pdf [file jz4c00826_si_001.pdf]

Supporting Information for

Chemical Aristocracy: He_3 Dication and Analogous Noble-Gas-Exclusive Compounds

Lucas Araujo[1], Felipe Fantuzzi[2*], Thiago M. Cardozo[1*]

[1]Instituto de Química, Universidade Federal do Rio de Janeiro, Av. Athos da Silveira Ramos 149, Rio de Janeiro 21941-909, Brazil

[2]School of Chemistry and Forensic Science, University of Kent, Park Wood Rd, Canterbury CT2 7NH, UK

Computational Details

Geometry optimization and vibrational frequency calculations. Geometry optimizations and vibrational frequency calculations were conducted using three distinct methodologies. These included Kohn–Sham DFT with the double-hybrid DSD-BYLYP functional[S1] for exchange and correlation, second-order Møller-Plesset perturbation theory (MP2), and coupled-cluster with singles, doubles and perturbative triples excitations (CCSD(T)). All calculations employed the augmented Dunning correlation consistent aug-cc-pVTZ basis set and were performed using the ORCA 4.2 software.[S2] Additionally, for He_3^{2+}, we conducted supplementary calculations based on average quadratic coupled-cluster (AQCC)[S3] with the aug-cc-pVQZ basis set for benchmarking purposes. Notably, the optimized geometries remained consistent across all employed methods, and the frequency analysis confirmed that all discussed species represent energy minima, exhibiting no imaginary vibrational frequencies.

Chemical bonding and charge analyses. To investigate the nature of the chemical bond within the true minimum energy structures, we employed spin-coupled generalized valence bond (SCGVB)[S4-S6] and quantum theory of atoms in molecules (QTAIM)[S7,S8] calculations. The SCGVB calculations were executed using the VB2000[S9] software as implemented in GAMESS (version September 30, 2022 R2).[S10] Both full SCGVB and its perfect-pairing approximation (SCGVB-PP)[S11,S12] were utilized in conjunction with the generalized product function energy partitioning (GPF-EP) method (vide infra), employing cc-pVTZ basis sets. The GPF-EP[S13] method facilitated the execution of the interference energy analysis (IEA) for He_3^{2+}, offering deeper insights into the covalent nature of its chemical bond. Complementing this, intrinsic bond orbitals (IBOs)[S14,S15] were generated at the PBE0/def2-QZVPP level of theory, expanding our understanding of the bond situation within the optimized molecules. IBO calculations, along with SCGVB and SCGVB-PP orbital depictions, were obtained using the IboView software. Mulliken[S16] and ChelpG[S17] charges and QTAIM topological indices were obtained using relaxed MP2 and coupled-cluster densities from natural orbitals and associated density matrix were generated with ORCA and processed with Multiwfn 3.8 software.[S18]

The GPF-EP method. The generalized product function (GPF)[S18] provides an approximate solution for the many-electrons problem. In a system comprising *N* electrons, these electrons are categorized and separated into groups, each exhibiting an average dependence akin to the self-consistent field equations of the Hartree-Fock (HF) method. The wavefunction takes the following form:

$$\Psi(\vec{x}_1, \ldots, \vec{x}_n) = \mathcal{A}\left[\Psi^{(1)}\left(\vec{x}_1, \ldots, \vec{x}_{n^{(1)}}\right)\Psi^{(2)}\left(\vec{x}_{\alpha+1}, \vec{x}_{\alpha+2}, \ldots, \vec{x}_{n^{(1)}+n^{(2)}}\right) \ldots\right] \quad (1)$$

where $\vec{x}_i$ represents the spatial and spin coordinates of the i-th electron. The superscripts (1), (2), ..., indicate the antisymmetrized wavefunctions of the electron groups with $n^{(1)}, n^{(2)}, \ldots, n^{(N)}$ electrons. Moreover, the wavefunctions and orbitals of each group are orthogonal. The antisymmetrizing operator $\mathcal{A}$ only contains permutations involving one electron from each group. The VB2000 software is based on the use of GPF wavefunctions, allowing users to select a specific description for each electron group, such as HF, classical VB, SCGVB, or CASVB. Specifically, in SCGVB-PP calculations, the core electrons are treated as an HF group, while the valence electrons are distributed into groups consisting of two electrons, described using the SCGVB approach.

The GPF-EP[51] method employs a GPF to express first and second-order reduced density matrices as interference and reference (quasi-classical) densities. For a single group, the expressions for the quasi-classical and interference densities are as follows:

$$\rho_{QC}^{\mu} = \sum_{j=1}^{N^{\mu}} \left[\phi_j^{\mu}(\vec{r})\right]^2 \quad (2)$$

$$\rho_I^{\mu} = \sum_{j,k}^{N^{\mu}} \langle j,k\rangle^{\mu} p(j|k) \quad (3)$$

where μ indicates the group under consideration, N^{μ} denotes the number of electrons in this group, $\langle j,k\rangle^{\mu}$ represents the interference density associated with the orbitals ϕ_j and ϕ_k, and $p(j|k)$ corresponds to the elements of the density matrix in the orbital basis set. Specifically:

$$\langle j,k\rangle^{\mu} = \phi_j^{\mu}(\vec{r})\phi_k^{\mu}(\vec{r}) - \frac{1}{2}\xi(j,k)\left\{\left[\phi_j^{\mu}(\vec{r})\right]^2 + \left[\phi_k^{\mu}(\vec{r})\right]^2\right\} \quad (4)$$

with $\xi(j,k)$ being the overlap integral between the orbitals ϕ_j and ϕ_k.

This approach can be extended to the pair density, resulting in a partition of the total energy of the system as follows:

$$E[tot] = E[ref] + E[X] + E[I] + E[II] \quad (5)$$

$E[ref]$ represents the total reference energy, E[I] and E[II] denote the first and second-order interference energies, while $E[X]$ accounts for the total intergroup exchange interaction, arising from the antisymmetry of the GPF. In other words, $E[X]$ introduces a symmetry correction to the reference energy due to the separation of the N-electron wavefunction into groups. The sum $E[ref] + E[X]$ corresponds to a (symmetry-corrected) quasi-classical contribution, and the sum $E[I] + E[II]$ corresponds to the interference contribution. This framework has found successful applications in describing chemical bond formation and the role of quantum interference in bonding, as evidenced by a substantial body of literature focused on various case studies.[S13,S19-S34]

Additional Results

Table S1. Calculated bond lengths (Å) using various methods with the aug-cc-pVTZ basis set.

	**DSD-BLYP**		**MP2**		**CCSD(T)**	
Molecule (Ng1-Ng2-Ng3)	Ng1-Ng2	Ng2-Ng3	Ng1-Ng2	Ng2-Ng3	Ng1-Ng2	Ng2-Ng3
He_3^{2+}	0.8786	0.8786	0.8783	0.8783	0.925	0.925
He_2Ne^{2+}	0.9513	1.1811	0.9559	1.2269	-	-
He_2Ar^{2+}	1.4076	1.3707	1.4718	1.3462	1.456	1.384
$HeNeAr^{2+}$	1.9077	1.7600	2.0668	1.7234	1.993	1.782
$HeArHe^{2+}$	1.5312	1.5312	1.5226	1.5226	1.545	1.545
$HeArNe^{2+}$	1.5562	1.8327	1.5524	1.8115	1.575	1.847
$HeAr_2^{2+}$	2.0607	2.0163	2.1283	2.0087	2.145	2.068
$NeHeAr^{2+}$	1.4950	1.4608	1.4739	1.4013	-	-
$NeArNe^{2+}$	1.8602	1.8602	1.8410	1.8410	1.874	1.874
$NeAr_2^{2+}$	2.1600	2.0418	2.1903	2.0284	2.215	2.088
Ar_3^{2+}	2.2889	2.2889	2.2931	2.2931	2.399	2.399

Table S2. Total Energy (a.u.), zero-point energy (a.u), vertical singlet-triplet gap (kcal/mol), calculated harmonic frequencies (cm^{-1}) and intensities (km/mol) at CCSD(T)/aug-cc-pVTZ, and bonding pattern according to Scheme 1.

Molecule	Singlet	ZPE	S-T gap	Frequencies (Intensities)	Bonding Pattern
He_3^{2+}	-6.755890671	0.012	148.38	942.94 (0.37), 942.94 (0.37), 1169.80 (0.00), 2207.86 (283.90) [951.61 (0.20), 951.61 (0.20), 1215.35 (0.00), 2235.80 (306.39)]*	I
He_2Ar^{2+}	-531.3049966	0.0049	52.66	265.22 (12.97), 265.23 (12.97), 429.19 (105.88), 1194.85 (262.15)	IV
$HeNeAr^{2+}$	-657.2322793	0.0021	14.01	95.33 (0.02), 95.34 (0.02), 253.34 (61.15), 476.91 (10.65)	II
$HeArHe^{2+}$	-531.3323271	0.0062	65.51	519.11 (8.61), 519.11 (8.61), 690.89 (264.48), 995.46 (0.00)	III
$HeArNe^{2+}$	-657.2636455	0.0045	50.79	361.54 (1.47), 361.54 (1.47), 477.66 (80.31), 783.56 (241.67)	II
$HeAr_2^{2+}$	-1055.679278	0.0021	46.93	122.69 (0.18), 122.70 (0.18), 279.95 (42.66), 378.42 (378.42)	IV
$NeArNe^{2+}$	-783.1941543	0.003	39.73	203.01 (2.89), 203.08 (2.89), 394.67 (0.11), 502.94 (203.13)	II
$NeAr_2^{2+}$	-1181.597576	0.0016	48.05	108.34 (1.29), 108.34 (1.29), 213.48 (59.16), 359.30 (31.95)	IV
Ar_3^{2+}	-1579.91865994	0.0014	35.75	104.02 (0.37), 104.02 (0.37), 118.12 (0.00), 289.29 (0.08)	I

*AQCC/aug-cc-pVQZ

Table S3. Mulliken and ChelpG atomic charges obtained using various methods with the aug-cc-pVTZ basis set.

**DSD-BLYP**	Mulliken			ChelpG		
Molecule(Ng1-Ng2-Ng3)	q(Ng1)	q(Ng2)	q(Ng3)	q(Ng1)	q(Ng2)	q(Ng3)
He_3^{2+}	0.632	0.737	0.632	0.751	0.498	0.751
He_2Ne^{2+}	0.574	0.666	0.760	0.706	0.409	0.885
He_2Ar^{2+}	0.168	0.302	1.530	0.240	0.220	1.539
$HeNeAr^{2+}$	0.059	0.468	1.473	0.147	0.382	1.470
$HeArHe^{2+}$	0.268	1.465	0.268	0.446	1.107	0.446
$HeArNe^{2+}$	0.247	1.430	0.323	0.430	1.054	0.515
$HeAr_2^{2+}$	0.029	1.008	0.963	0.190	0.755	1.055
$NeHeAr^{2+}$	0.337	0.293	1.371	0.450	0.138	1.412
$NeArNe^{2+}$	0.320	1.360	0.320	0.501	0.998	0.501
$NeAr_2^{2+}$	0.111	0.983	0.906	0.270	0.705	1.025
Ar_3^{2+}	0.648	0.703	0.648	0.784	0.431	0.784
**MP2**	Mulliken			ChelpG		
Molecule(Ng1-Ng2-Ng3)	q(Ng1)	q(Ng2)	q(Ng3)	q(Ng1)	q(Ng2)	q(Ng3)
He_3^{2+}	0.665	0.670	0.665	0.753	0.493	0.753
He_2Ne^{2+}	0.608	0.635	0.757	0.727	0.388	0.884
He_2Ar^{2+}	0.113	0.326	1.561	0.192	0.244	1.564
$HeNeAr^{2+}$	0.011	0.495	1.494	0.093	0.418	1.488
$HeArHe^{2+}$	0.264	1.472	0.264	0.444	1.112	0.444
$HeArNe^{2+}$	0.242	1.433	0.325	0.424	1.055	0.521
$HeAr_2^{2+}$	0.013	1.012	0.975	0.169	0.767	1.064
$NeHeAr^{2+}$	0.272	0.329	1.399	0.394	0.156	1.450
$NeArNe^{2+}$	0.320	1.360	0.320	0.503	0.994	0.503
$NeAr_2^{2+}$	0.083	0.995	0.922	0.241	0.719	1.040
Ar_3^{2+}	0.669	0.661	0.669	0.799	0.403	0.799
**CCSD(T)**	Mulliken			ChelpG		
Molecule(Ng1-Ng2-Ng3)	q(Ng1)	q(Ng2)	q(Ng3)	q(Ng1)	q(Ng2)	q(Ng3)
He_3^{2+}	0.707	0.585	0.707	0.769	0.462	0.769
He_2Ar^{2+}	0.122	0.325	1.553	0.184	0.259	1.557
$HeNeAr^{2+}$	0.012	0.490	1.498	0.089	0.424	1.487

$HeArHe^{2+}$	0.250	1.500	0.250	0.425	1.150	0.425
$HeArNe^{2+}$	0.220	1.469	0.310	0.393	1.108	0.499
$HeAr_2^{2+}$	0.009	1.006	0.985	0.142	0.791	1.068
$NeArNe^{2+}$	0.306	1.388	0.306	0.470	1.060	0.470
$NeAr_2^{2+}$	0.060	1.018	0.922	0.214	0.768	1.018
Ar_3^{2+}	0.656	0.688	0.656	0.769	0.461	0.769

Table S4. Dissociation paths calculated at the CCSD(T)/aug-cc-pVTZ level with and without zero-point energy (ZPE). For He^{+}, the full configuration interaction (FCI) method was employed with the same basis set.

Molecule	Dissociation Paths	Without ZPE	With ZPE
**He_3^{2+}**	**$He + 2\ He^{1+}$**	-89.45	-96.97
	$He^{1+} + He_2^{1+}$	-146.00	-151.10
	$He + He_2^{2+}$	111.77	108.89
**He_2Ar^{2+}**	$He + He^{1+} + Ar^{1+}$	-43.63	-46.71
	$2\ He^{1+} + Ar$	162.14	159.06
	$He^{1+} + HeAr^{1+}$	-44.75	-47.61
	$HeHe^{1+} + Ar^{1+}$	-100.18	-100.84
	$He + HeAr^{2+}$	6.30	4.99
	$He_2(2+) + Ar$	363.36	364.93
**$HeNeAr^{2+}$**	$He + Ne^{1+} + Ar^{1+}$	-104.92	-106.23
	$He^{1+} + Ne + Ar^{1+}$	-34.04	-35.36
	$He^{1+} + Ne^{1+} + Ar$	100.69	99.38
	$HeNe^{1+} + Ar^{1+}$	-119.85	-120.18
	$He^{1+} + NeAr^{1+}$	-36.55	-37.68
	$HeAr^{1+} + Ne^{1+}$	-106.12	-107.22
	$HeNe^{1+} + Ar$	303.70	304.18
	$He + NeAr^{1+}$	2.42	1.87
	$HeAr^{1+} + Ne$	15.86	16.31
**$HeArHe^{2+}$**	$He + He^{1+} + Ar^{1+}$	-26.48	-30.37
	$2\ He^{1+} + Ar$	179.29	175.40

	$He_2^{1+} + Ar^{1+}$	-83.03	-84.50
	$He^{1+} + HeAr^{1+}$	-27.60	-31.27
	$He_2^{2+} + Ar$	380.51	381.27
	$HeAr^{2+} + He$	23.45	21.33
**$HeArNe^{2+}$**	$He + Ne^{1+} + Ar^{1+}$	-85.25	-88.08
	$He^{1+} + Ne + Ar^{1+}$	-14.38	-17.21
	$He^{1+} + Ne^{1+} + Ar$	120.36	117.53
	$HeNe^{1+} + Ar^{1+}$	-100.17	-102.02
	$He^{1+} + NeAr^{1+}$	-16.87	-19.51
	$HeAr^{1+} + Ne^{1+}$	-86.44	-89.05
	$HeNe^{2+} + Ar$	323.38	322.35
	$He + NeAr^{2+}$	22.11	20.03
	$HeAr^{2+} + Ne$	35.54	34.47
**$HeAr_2^{2+}$**	$He + 2\ Ar^{1+}$	-107.51	-108.80
	$He^{1+} + Ar + Ar^{1+}$	98.26	96.97
	$He^{1+} + Ar_2^{1+}$	68.08	67.22
	$HeAr^{1+} + Ar^{1+}$	-108.64	-109.70
	$He + Ar_2^{2+}$	3.41	2.67
	$HeAr^{2+} + Ar$	148.19	148.67
**$NeArNe^{2+}$**	$Ne + Ne^{1+} + Ar^{1+}$	-73.73	-75.60
	$2\ Ne^{1+} + Ar$	61.11	59.25
	$Ne_2^{1+} + Ar^{1+}$	-105.89	-106.91
	$Ne^{1+} + NeAr^{1+}$	-76.21	-77.89
	$NeAr^{2+} + Ne$	33.69	32.59
**$NeAr_2^{2+}$**	$Ne + 2\ Ar^{1+}$	-103.59	-104.57
	$Ne^{1+} + Ar + Ar^{1+}$	31.25	30.28
	$Ne^{1+} + Ar_2^{1+}$	1.07	0.53
	$NeAr^{1+} + Ar^{1+}$	-106.07	-106.85
	$Ne + Ar_2^{2+}$	7.33	6.91
	$NeAr^{2+} + Ar$	138.68	138.47

**Ar_3^{2+}**	$Ar + 2Ar^{1+}$	-50.27	-51.15
	$Ar^{1+} + Ar_2^{1+}$	-80.45	-80.90
	$Ar + Ar_2^{2+}$	60.65	60.32

Fig. S1: SCGVB-PP orbitals at the SCGVB-PP/aug-cc-pVTZ level of theory. For Ar_3^{2+}, four valence electrons were treated in one GVB group and the remaining electrons are placed in a Hartree-Fock group. The numbers in black represent the bond lengths at the CCSD(T)/aug-cc-pVTZ level, while the numbers in red denote the ChelpG atomic charges.

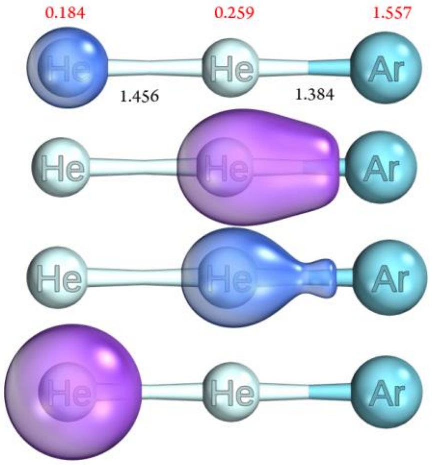

HeHeAr²⁺

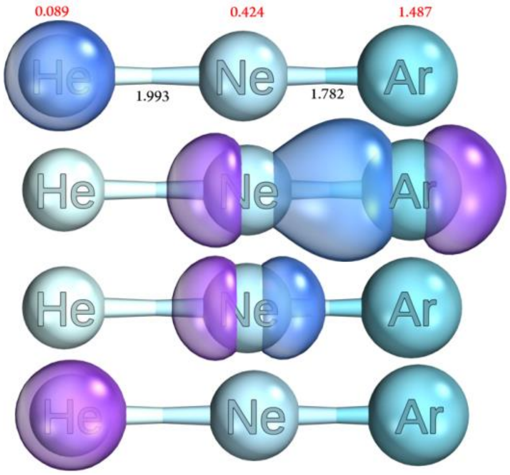

HeNeAr²⁺

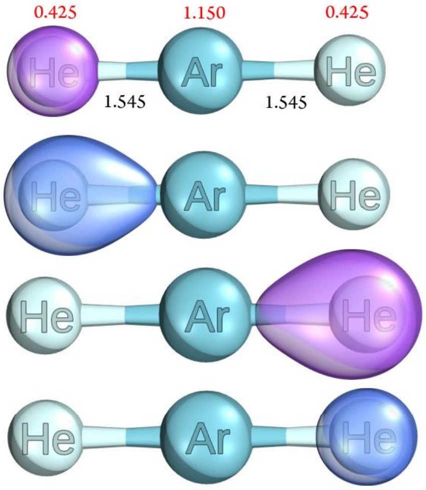

HeArHe²⁺

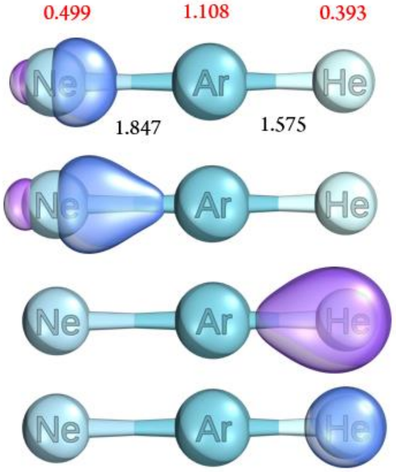

NeArHe²⁺

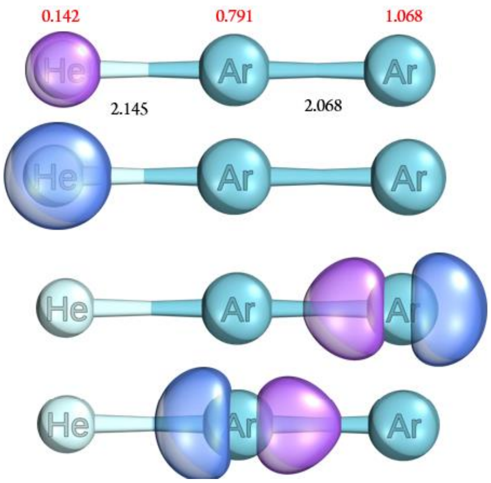

HeArAr^{2+}

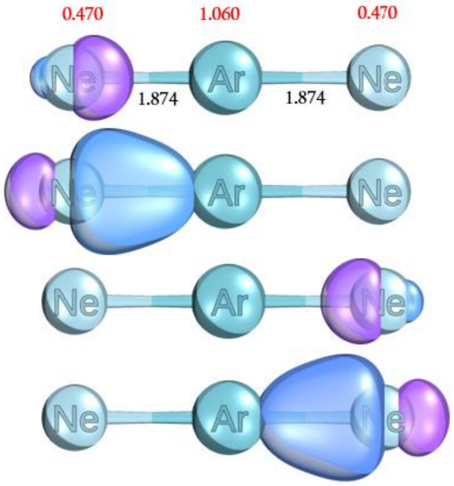

NeArNe^{2+}

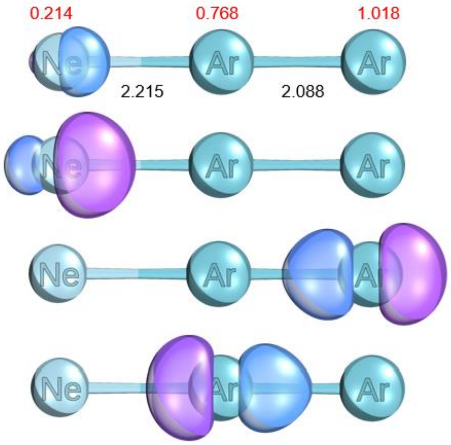

NeArAr^{2+}

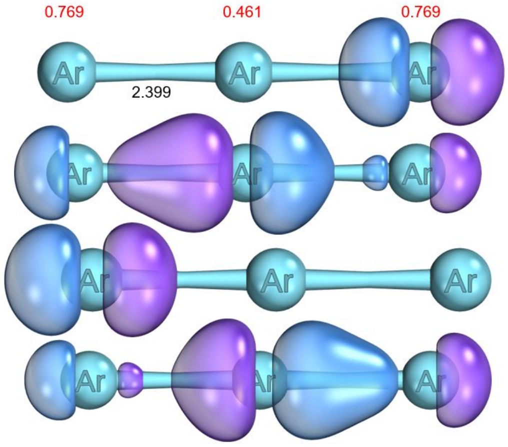

ArArAr^{2+}

Fig. S2: Laplacian of the electron density of all minimum structures at the CCSD(T)/aug-cc-pVTZ level. Bond critical points (BCPs) are shown as red dots.

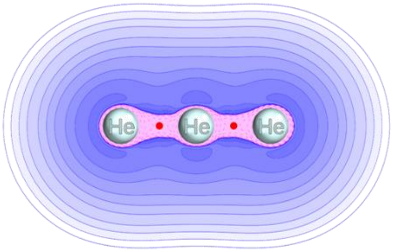

He_3^{2+}

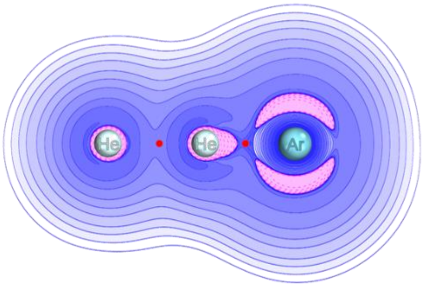

He_2Ar^{2+}

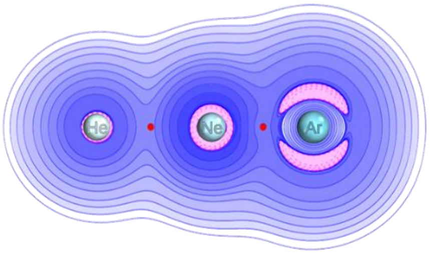

$HeNeAr^{2+}$

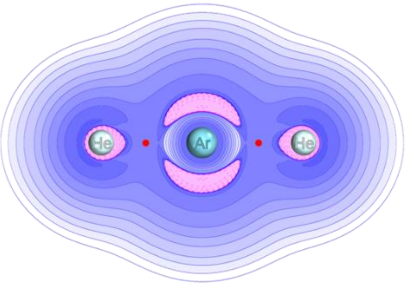

$HeArHe^{2+}$

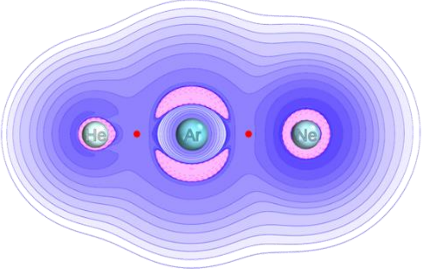

$HeArNe^{2+}$

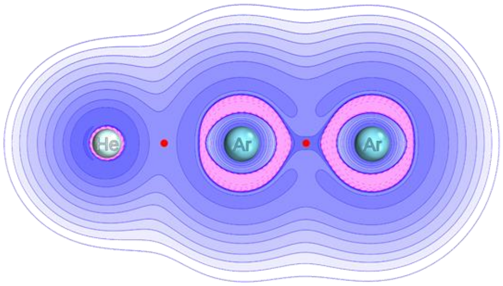

$HeAr_2^{2+}$

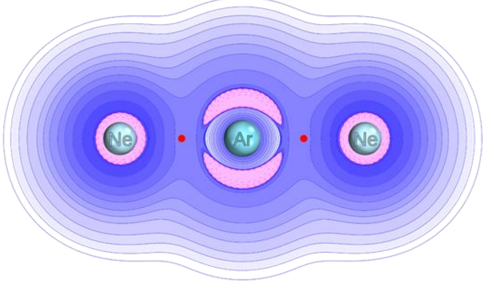

$NeArNe^{2+}$

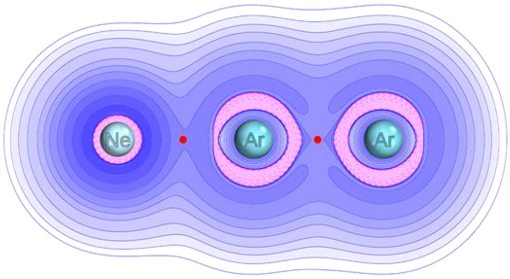

$NeAr_2^{2+}$

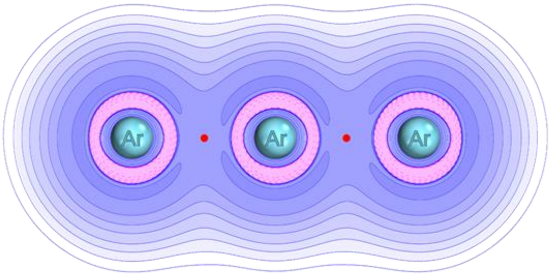

Ar_3^{2+}

Fig. S3: IBOs (PBE0/def2-QZVPPD) from optimized geometries at the CCSD(T)/aug-cc-pVTZ level of theory.

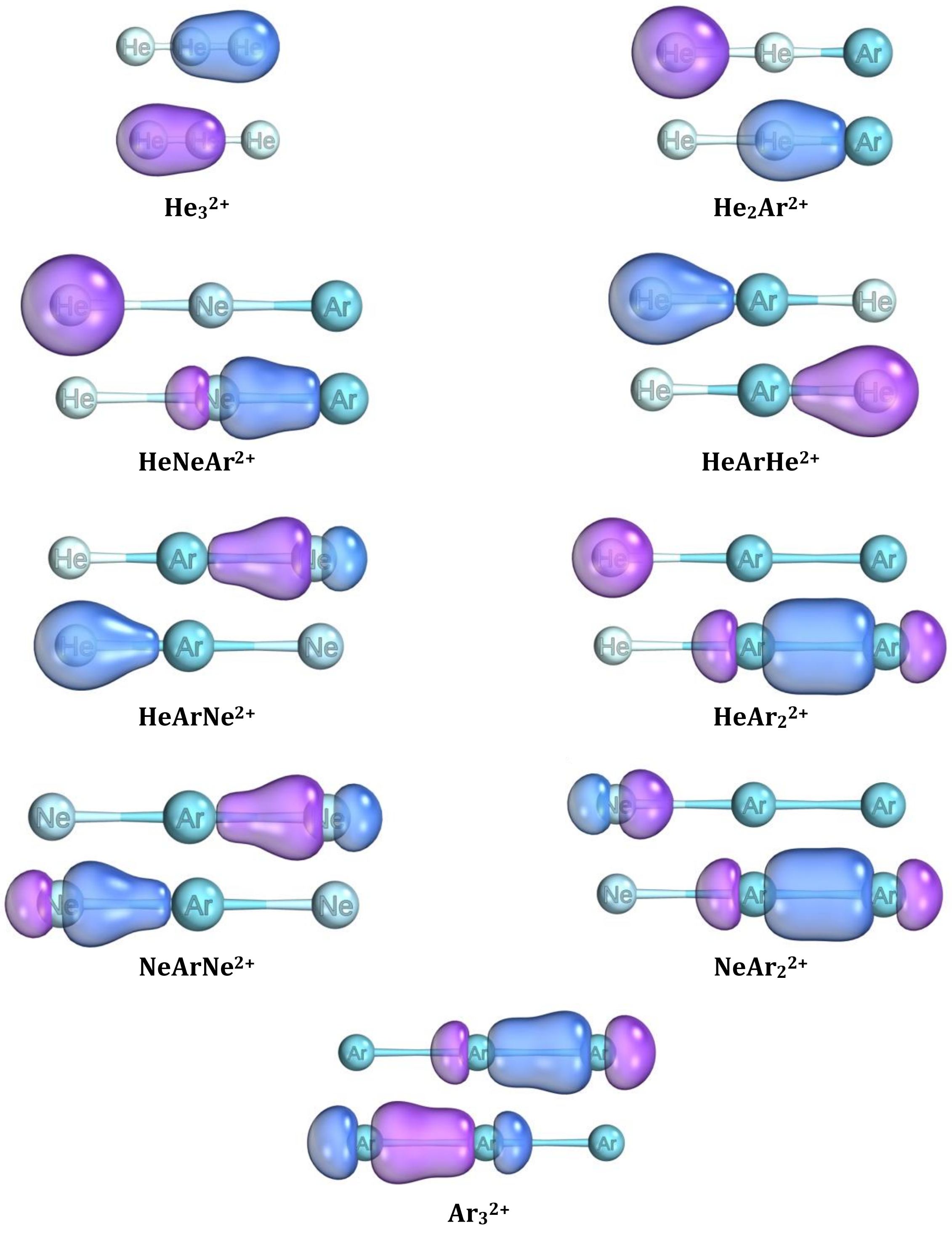

References

(S1) Kozuch, S., Gruzman, D.; Martin, J. M. L. DSD-BLYP: A general purpose double hybrid density functional including spin component scaling and dispersion correction. *J. Phys. Chem. C*. **2010**, *114*, 20801–20808.
(S2) Neese, F. Software update: the ORCA program system, version 4.0. *Wiley Interdiscip. Rev. Comput. Mol. Sci*. **2018**, *8*, e1327.
(S3) Szalay, P. G.; Bartlett, R. Multi-reference averaged quadratic coupled-cluster method: A size-extensive modification of multi-reference CI. *J. Chem. Phys. Lett*. **1993**, *214*, 481–488.
(S4) Gerratt, J.; Lipscomb, W. N. Spin-coupled wave functions for atoms and molecules. *Proc. Natl. Acad. Sci*. **1968**, 59, 332–335.
(S5) Goddard III, W. A. Improved quantum theory of many-electron systems. I. Construction of eigenfunctions of S^2 which satisfy Pauli's principle. *Phys. Rev*. **1967**,*157*, 73–80.
(S6) Goddard III, W. A. Improved quantum theory of many-electron systems. II. The basic method. *Phys. Rev*. **1967**, *157*, 81–93.
(S7) Bader, R. F. W.; Essén, H. The characterization of atomic interactions. *J. Chem. Phys*. **1984**, *80*, 1943–1960.
(S8) Bader, R. F. W. Atoms in molecules: A quantum theory, Oxford University Press, Oxford, **1990**.
(S9) Li, J.; McWeeny, R. VB2000: Pushing valence bond theory to new limits. *Int. J. Quantum Chem*. **2002**, *89*, 208-216.
(S10) Barca, G. M. et al. Recent developments in the general atomic and molecular electronic structure system. *J. Chem. Phys*. **2020**, *152*, 154102.
(S11) Bobrowicz, F. W.; Goddard III, W. A. The self-consistent field equations for generalized valence bond and open-shell Hartree-Fock wave functions. in Methods of Electronic Structure Theory (ed. Schaefer, H. F.), Springer, New York, 3, pp. 79–127, **1977**.
(S12) Faglioni, F.; Goddard III, W. A. GVB–RP: A reliable MCSCF wave function for large systems. *Int. J. Quantum Chem*. **1999**, *73*, 1–22.
(S13) Cardozo, T. M.; Nascimento, M. A. C. Energy partitioning for generalized product functions: The interference contribution to the energy of generalized valence bond and spin coupled wave functions. *J. Chem. Phys*. **2009**, *130*, 104102.
(S14) Knizia, G. Intrinsic atomic orbitals: An unbiased bridge between quantum theory and chemical concepts. *J. Chem. Theory Comput*. **2013**, *9*, 4834–4843.
(S15) Knizia, G.; Klein, J. E. Electron flow in reaction mechanisms–Revealed from first principles. *Angew. Chem. Int. Ed*. **2015**, *54*, 5518–5522.
(S16) Mulliken, R. S. Electronic population analysis on LCAO–MO molecular wave functions. I. *J. Chem. Phys*. **1955**, *23*, 1833–1840.
(S17) Breneman, C. M.; Wiberg, K. B. Determining atom-centered monopoles from molecular electrostatic potentials. The need for high sampling density in formamide conformational analysis. *J. Comput. Chem*. **1990**, *11*, 361–373.
(S18) Lu, T.; Chen, F. Multiwfn: A multifunctional wavefunction analyzer. *J. Comput. Chem*. **1990**, *33*, 580–592.
(S19) Sousa, D. W. O. D.; Nascimento, M. A. C. Are one-electron bonds any different from standard two-electron covalent bonds?. *Acc. Chem. Res*. **2017**, *50*, 2264–2272.
(S20) Fantuzzi, F.; Sousa, D. W. O. D.; Nascimento, M. A. C. The nature of the chemical bond from a quantum mechanical interference perspective. *ChemistrySelect* **2017**, *2*, 604–619.
(S21) Ruedenberg, K. The physical nature of the chemical bond. *Rev. Mod. Phys*. **1962**, *34*, 326–376.
(S22) Fantuzzi, F.; Cardozo, T. M.; Nascimento, M. A. C. On the metastability of doubly charged homonuclear diatomics. *Phys. Chem. Chem. Phys*. **2017**, *19*, 19352–19359.
(S23) Feinberg, M. J.; Ruedenberg, K. Paradoxical role of the kinetic-energy operator in the formation of the covalent bond. *J. Chem. Phys*. **1971**, *54*, 1495–1511.
(S24) Ruedenberg, K.; Schmidt, M. W. Why does electron sharing lead to covalent bonding? A variational analysis. *J. Comput. Chem*. **2007**, 28, 391–410.

(S25) Ruedenberg, K.; Schmidt, M. W. Physical understanding through variational reasoning: Electron sharing and covalent bonding. *J. Phys. Chem. A*. **2009**, *113*, 1954–1968.
(S26) Schmidt, M. W.; Ivanic, J.; Ruedenberg, K. Covalent bonds are created by the drive of electron waves to lower their kinetic energy through expansion. *J. Chem. Phys*. **2014**, *140*, 204104.
(S27) Kutzelnigg, W. The physical mechanism of the chemical bond. *Angew. Chem., Int. Ed. Engl*. **1973**, 12, 546–562.
(S28) Wilson Jr, C. W.; Goddard III, W. A. Exchange kinetic energy, contragradience, and chemical binding. *Chem. Phys. Lett*. **1970**, *5*, 45–49.
(S29) Wilson Jr, C. W.; Goddard III, W. A. The role of kinetic energy in chemical binding: I. The nonclassical or exchange kinetic energy. *Theor. Chim. Acta*. **1972**, *26*, 195–210.
(S30) Wilson Jr, C. W.; Goddard III, W. A. The role of kinetic energy in chemical binding: II. Contragradience. *Theor. Chim. Acta*. **1972**, *26*, 211–230.
(S31) Cardozo, T. M.; Nascimento, M. A. C. Chemical bonding in the N2 molecule and the role of the quantum mechanical interference effect. *J. Phys. Chem. A*. **2009**, *113*, 12541–12548.
(S32) Cardozo, T. M.; Freitas, G. N.; Nascimento, M. A. C. Interference effect and the nature of the π-bonding in 1,3-butadiene. *J. Phys. Chem. A*. **2010**, *114*, 8798–8805.
(S33) Cardozo, T. M.; Sousa, D. W. O. D.; Fantuzzi, F.; Nascimento, M. A. C. The chemical bond as a manifestation of quantum mechanical interference: Theory and applications of the interference energy analysis using SCGVB wave functions. in Comprehensive Computational Chemistry 1, 552–588, Elsevier, Amsterdam, **2024**.
(S34) Levine, D. S.; Head-Gordon, M. Clarifying the quantum mechanical origin of the covalent chemical bond. *Nat. Commun*. **2020**, *11*, 4893.
